# Mortality benefits of population-wide adherence to national physical activity guidelines: a prospective cohort study

**DOI:** 10.1007/s10654-014-9965-5

**Published:** 2014-11-07

**Authors:** Gráinne Long, Clare Watkinson, Søren Brage, Jerry Morris, Bill Tuxworth, Peter Fentem, Simon Griffin, Rebecca Simmons, Nicholas Wareham

**Affiliations:** 1 MRC Epidemiology Unit, Box 285 Institute of Metabolic Science, University of Cambridge School of Clinical Medicine, Cambridge Biomedical Campus, Cambridge, CB2 0QQ UK; 2 London School of Hygiene and Tropical Medicine, London, UK; 3 Department of Sport and Exercise Science, University of Birmingham, Birmingham, UK; 4 Department of Medicine, University of Nottingham, Nottingham, UK

**Keywords:** Physical activity, Physical activity guidelines, All-cause mortality, Population health promotion, Attributable fraction

## Abstract

We quantified the mortality benefits and attributable fractions associated with engaging in physical activity across a range of levels, including those recommended by national guidelines. Data were from the Allied Dunbar National Fitness Survey, a population-based prospective cohort comprising 1,796 male and 2,122 female participants aged 16–96 years, randomly selected from 30 English constituencies in 1990. Participants were tagged for mortality at the Office for National Statistics. Cox multivariable regression quantified the association between self-reported achievement of activity guidelines—150 min of at least moderate activity per week, equivalent here to 30 or more 20-min episodes of at least moderate activity per month—and mortality adjusting for age, sex, smoking status, social class, geographical area, anxiety/depression and interview season. There were 1,175 deaths over a median (IQR) of 22.9 (3.9) years follow-up; a mortality rate of 15.2, 95 % confidence interval (CI) 14.4–16.1 per 1,000 person years. Compared with being inactive (no 20-min bouts per month), meeting activity guidelines (30+ bouts) was associated with a 25 % lower mortality rate, adjusting for measured confounders. If everyone adhered to recommended-, or even low-activity levels, a substantial proportion of premature mortality might be avoided (PAF, 95 % CI 20.6, 6.9–32.3 and 8.9, 4.2–13.4 %, respectively). Among a representative English population, adherence to activity guidelines was associated with significantly reduced mortality. Efforts to increase population-wide activity levels could produce large public health benefits and should remain a focus of health promotion efforts.

**Electronic supplementary material:**

The online version of this article (doi:10.1007/s10654-014-9965-5) contains supplementary material, which is available to authorized users.

## Introduction

The Department of Health has recently set out an ambitious call for action aimed at reducing the rate of premature mortality in England to reach levels among the lowest in Europe by 2020 [1]. Through a combined strategy of prevention, early diagnosis and treatment, it is hoped that a step-change in the health of the nation will be achieved, with concomitant reductions in premature mortality. Preventative public health approaches that target key modifiable risk factors, such as physical inactivity, are a vital tool in the fight against premature death [2]. The physical activity guidelines of leading global public health agencies have converged on recommending a minimum amount of 150 min per week of at least moderate intensity exercise to achieve general health benefits [3–6]. Evidence to support the health benefits of performing activity in multiple bouts throughout the week is growing, and should be considered where possible [7]. Previous studies have shown that a minority of men and women in the UK report meeting these minimum recommendations for physical activity [8, 9]. Few studies have directly assessed whether meeting these recommended activity levels reduces mortality risk and those that have, focused solely on leisure time activity [10, 11]. To our knowledge, no study has considered total moderate and vigorous-intensity physical activity over all domains and directly assessed whether meeting physical activity guidelines reduces mortality risk. A better understanding of the relationship between adherence to physical activity recommendations and mortality is needed to inform public health efforts that encourage individuals to meet activity guidelines and to determine the priority that should be given to them.

In 1990, the first national survey of fitness was carried out in England which included assessment of the intensity, frequency and duration of total daily physical activity in a representative sample of English men and women aged 16–96 years [12, 13]. We aimed to examine whether achieving the recommended activity guidelines of 150 min of at least moderate intensity activity per week was associated with reduced all-cause mortality rates over 22 years of follow-up in this large population-based prospective cohort. We also describe the association between mortality and participation in different amounts of activity. Finally, we estimate how many premature deaths might be avoided in the entire population, and in the subgroup of inactive individuals, if they achieved a range of different physical activity levels, including those recommended in guidelines.

## Methods

### Study design

The Allied-Dunbar National Fitness Survey (ADNFS) was conducted in a representative sample of English adults between February and November 1990 (http://discover.ukdataservice.ac.uk/catalogue?sn=3303 [14]). Probability sampling procedures randomly selected 30 English parliamentary constituencies out of 523. Within each constituency, 200 addresses were randomly chosen from the electoral register and one adult per household chosen at random [15]. Out of the 5,698 men and women aged 16 and over approached for survey, 4,316 participated in ADNFS; a 76 % response rate. Due to the sampling procedure which focused on the adult population in households, non-responders tended to be younger and from lower social classes. However, differences were small and ADNFS participants were representative of the age and sex distribution of the English population at that time [12, 13]. Interviewers from the Social Survey Division of the Office of Population Censuses and Surveys (OPCS) conducted structured interviews in participants’ homes. Information on socio-demographic characteristics, physical activity, health and lifestyle were collected by questionnaire at baseline interview [14]. The survey protocol was approved by the local Research Ethical Committees of each Health District involved [16].

### Outcome measurement

The main outcome measure was all-cause mortality. All ADNFS participants were tagged for mortality and migration at the Office of National Statistics (ONS) from their survey date in 1990 to 14th May 2014 (n = 1,175 deaths and n = 145). Deaths were coded into four categories (cardiovascular, cancer, suicide/violence/accidental, and other) based on the classification of the underlying cause of death against the International Classification of Diseases, tenth edition (ICD-10). Classifications for CVD deaths were defined by ICD codes in the range I00–I99, cancer deaths by codes in the range C00–D48, and suicide/violence/accidental deaths by codes in the range V01–Y98. This classification was independently done by an assessor masked to exposure data. A 5 % sample was randomly selected and independently classified by a second researcher, with 100 % agreement.

### Explanatory variables and covariates

The primary exposure was the number of occasions of self-reported 20-min episodes of moderate to vigorous physical activity in the past month (activity bouts). The ADNFS questionnaire was designed to capture the frequency (number of times in past month), duration (length of all activity engaged in lasting at least 1 min) and intensity (scored according to published energy costs [17–21]) of all activity engaged in and has been validated against walking speed and stair climbing [22]. At the time of the ADNFS survey (1990), three episodes of at least moderate activity of 20 min duration per week were recommended for maintaining/improving cardio-respiratory fitness and provide the rationale for producing a summary of current activity based on the number of occasions of moderate to vigorous activity of at least 20 min duration for each main activity type [14]. Information on bouts activity <20 min in length was not available to us. Participants were classified according to the range, frequency and intensity of self-reported physical activity bouts lasting at least 20 min over the 4 weeks prior to interview. Habitual activities comprised all sports and recreation, transportation, home activities and occupation, and was summarised into three energy bands; vigorous: ≥7.5 kcal/min (approximately ≥6.5 METs), moderate: 5–7.49 kcal/min (4–6.49 METs), and light: 2–4.9 kcal/min (1–3.99 METs) [14]. A habitual physical activity variable was derived based on the number of 20-min bouts of moderate to vigorous activity (>5 kcal/min; approximately >4 METs) in the past month, referred to here as physical activity bout. Current guidelines recommend at 150 min of at least moderate activity per week [9] and as the reference period used in the ADNFS study to assess current activity was past 4 weeks, recommended levels equate to 600 min of at least moderate activity per month. Thus, a categorical habitual activity measure was derived based on the number of 20-min physical activity bouts achieved, where the inactives reported 0 bouts, low actives reported 1–14, moderate actives reported 15–29, and actives—those meeting physical activity guidelines, here 30 bouts of 20 min—reported 30+ bouts per month, respectively.

A lifetime physical activity variable was collected at baseline and classified participants according to the proportion of their life spent regularly active in sports and exercise (participating in sports/recreation at least once a week, for at least 2 months of the year) since 14 years [23]. A lifetime participation proportion was calculated for every sports and exercise activity as previously published [23], by dividing the number of years of regular participation since age 14, by the current age minus 14 years. The decision to use the 14 year cut off, to restrict PA to only sports and recreational activities and to define regular lifetime activity of once a week for at least 2 months a year was based on early evaluation work by the ADNFS study team. They found inconsistencies in the reporting of childhood (<14 years), school curriculum linked activities and the frequency of lifetime activity (see ADNFS technical report [14].

Interviewers collected information on date of interview, socio-demographic characteristics (age, sex, occupation and marital status), regional health authority (RHA; NHS administrative units between 1974 and 1996) other lifestyle habits (smoking status and alcohol consumption) and prevalent disease at time of interview (stroke/MI, cancer). Socio-economic categories were assigned on the basis of occupation, according to the 1980 Registrar-General’s OPCS classification and comprised: (I) professional, (II) intermediate, (III) skilled, (IV) partly skilled, (V) unskilled and (VI) unclassified. Smoking (smokers, ex-smokers and non-smokers) and alcohol consumption (lights, moderate, heavy, none) were self-reported. BMI was recorded for a sub-sample of participants (n = 2,708/3,918) using a calibrated digital weighing scale and a metal stadiometer. Overweight was defined as 25 kg/m^2^ ≤ BMI < 30 kg/m^2^ and obesity as BMI ≥ 30 kg/m^2^, according to WHO criteria [24].

### Statistical analyses

Baseline characteristics were summarised separately according to survival status using means (SD) and percentages, and differences were examined using logistic regression. Individuals with missing data for an exposure of interest were included in all analyses not involving that specific exposure. To assess the nature of the relationship between activity and mortality, tests for departure from linear trend comprised a model including both categorical and log-linear terms for physical activity, followed by a Wald test for joint effect of categorical terms. A Cox regression model for the log hazard of death as a function of a restricted cubic spline for bouts of activity was fit to the data.

Cox proportional hazards regression was used to estimate the hazard ratios and corresponding 95 % confidence intervals (CI) for the association between meeting minimum activity guidelines [9] (here 30 or more 20-min bouts per month), lifetime physical activity, and all-cause mortality. Age is a strong determinant of mortality risk [25] and was used as the underlying time-scale for all models. Person-time for each participant was calculated from age at ADNFS interview to age at death or the study censor date (14th May 2014), whichever came first. A step-wise forward regression approach assessed the strength of the association between each variable and mortality, and overall model fit. Only those variables improving model fit were included in final models. Model 1 adjusted for age, sex and smoking status; known strong risk factors for mortality. Model 2 additionally adjusted for social class, geographical area, anxiety/depression and season of interview. Likelihood ratio tests (LRT) compared models with and without potential predictor variables. Interactions between physical activity and sex, social class and smoking status were examined via LRT.

To ascertain whether physical activity mediates its effects on mortality through BMI, Model 2 was repeated with and without BMI as a covariate and the percentage change in HR associated with mortality risk for physical activity variables was assessed. To assess bias from antecedent disease, sensitivity analyses were conducted omitting [1] those with a self-report of stroke, heart attack (MI) or cancer at baseline (n = 258), [2] deaths occurring within 5 years after interview (n = 31) and [3] those who were underweight (BMI < 18.5 kg/m^2^, n = 43). To assess the influence of migration on effect estimates, sensitivity analyses considering person-time before emigration were carried out, which censored individuals at date of first emigration [26], where available. Proportional hazards assumption was formally tested using the Schoenfeld and scaled Schoenfeld residuals. To estimate how much premature mortality could be prevented if all inactive individuals became low active, moderately active or active, the population attributable fraction (PAF) was calculated [27], by subtracting the marginal mean between–scenario hazard ratio and its confidence limits from 1 and adjusting for all known measured confounders (Model 2). The PAF for all-cause mortality associated with incremental increases in activity bouts were also calculated for the population as a whole, as well as stratified by sex (Supplementary Table 2). Statistical significance was set at a level of *p* < 0.05. Data was analysed using STATA version 13.0 (Stata, College Station, TX, USA).

## Results

Among the 3,918 participants, the median age was 45 years (range 16–96 years old). The majority were female (54 %), white (97 %), married (62 %), and belonged to higher social classes (45 and 25 % intermediate or professional occupations, respectively). 46 % were overweight or obese, 31 % were current smokers and 30 % reported a moderate or heavy intake of alcohol. Individuals who were not tracked by the Office of National Statistics were more likely belong a lower socioeconomic class (social class: χ_5_^2^ = 28.3, *p* ≤ 0.001), but were similar with respect to other baseline variables (data not shown). The median number (IQR) of 20-min bouts of moderate to vigorous activity in the preceding month was 8 [19] and only 15 % achieved guideline activity levels (20+ bouts per month). 20 % of individuals did not engage in any 20 min episodes of physical activity (inactive: 0 bouts per month) and 65 % engaged in some, but not guideline levels of activity (low and moderate active: 1–14; and 15–29 bouts per month, respectively). 34 % reported spending less than half of their lifetime engaged in regular sports and exercise activities from 14 years of age. Table 1 shows baseline characteristics by vital status. Participants were followed for a median (IQR) of 22.9 (3.9) years, giving 77,289 person-years at risk (PYR). During this time, 145 migrated and 1,175 participants died; a mortality rate of 15.2 (95 % CI 14.4–16.1) per 1,000 PYR.

**Table 1:** Association between demographic and lifestyle characteristics and all-cause mortality; the Allied Dunbar National Fitness Survey (n = 3,918, 1990–2013)

Characteristic	Participant N		Mortality odds ratio^a^	*p*_*trend*_
	All	Deceased (%)	OR (95 % CI)	
*Age* (*years*)				
16–29	891	24 (2.7)	–	–
30 to <45	1,008	63 (6.3)	–	
≥45 to <60	913	220 (24.1)	–	
≥60–96	1,106	868 (78.5)	–	
*Sex*				
Women	2,122	608 (28.7)	–	–
Men	1,796	567 (31.6)	–	
*SES* ^b^				
I	218	37 (17.0)	1	0.005
II	982	261 (26.6)	1.03 (0.73–1.46)	
III	1,775	534 (30.1)	1.26 (0.90–1.76)	
IV	645	220 (34.1)	1.36 (0.96–1.94)	
V	209	90 (43.1)	1.67 (1.06–2.31)	
VI	89	33 (37.1)	1.20 (0.75–1.94)	
*Marital status*				
Single	756	88 (11.6)	1	<0.001
Married	2,430	671 (27.6)	0.63 (0.49–0.79)	
Other^c^	730	415 (56.9)	0.74 (0.58–0.95)	
*BMI* (*range in kg/m* ^2^)^d^				
Healthy weight (14.9–24.9)	1,470	255 (17.4)	1	0.04
Overweight (25–29.9)	947	265 (28.0)	1.10 (0.93–1.31)	
Obese (30–49.9)	291	102 (35.1)	1.36 (1.08–1.72)	
*Habitual physical activity*				
Inactive (0 bouts/month)	787	492 (62.5)	1	<0.001
Low active (1–14 bouts/month)	1,773	460 (25.9)	0.77 (0.67–0.88)	
Moderate active (15–29 bouts/month)	778	147 (18.9)	0.78 (0.64–0.95)	
Active (30+ bouts/month)	574	76 (13.2)	0.71 (0.55–0.92)	
*Lifetime physical activity* ^e^				
0–24.9 %	523	154 (29.5)	1	0.12
25–49.9 %	548	112 (20.4)	0.87 (0.68–1.12)	
50–74.9 %	605	113 (18.7)	0.83 (0.64–1.05)	
75 %+	1,485	238 (16.0)	0.84 (0.69–1.04)	
*Smoking*				
Non-smoker	1,917	532 (27.8)	1	<0.001
Ex-smoker	791	276 (34.9)	1.52 (1.30–1.77)	
Current smoker	1,210	367 (30.3)	2.00 (1.74–2.30)	
*Alcohol intake*				
Light	2,373	698 (29.4)	1	0.001
Moderate	977	207 (21.2)	1.10 (0.94–1.29)	
Heavy	98	21 (21.4)	1.11 (0.72–1.72)	
Abstain	186	171 (91.9)	2.00 (0.89–1.28)	

Hazard ratios for continuous/categorical exposures represent the odds of mortality per 1 unit/categorical increase in the exposure

^a^Adjusted for age and sex

^b^According to the 1980 Registrar-General’s Office of Population Censuses and Surveys classification

^c^Divorced, separated, widowed

^d^Definition of healthy weight = BMI < 25 kg/m^2^; Overweight = 25 kg/m^2^ ≤ BMI < 30 kg/m^2^ and Obesity = BMI ≥ 30 kg/m^2^ [24]

^e^Based on the proportion of life spent active since 14 years

### Mortality benefits of being physically active (Table 2)

**Fig. 1:**
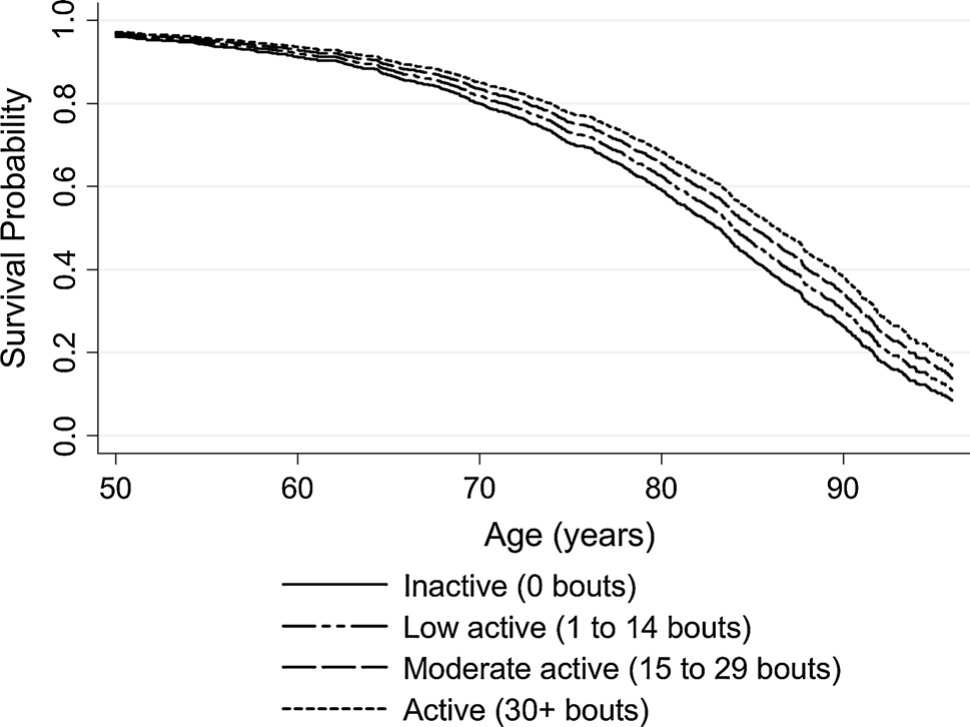
Survival probability from age 50 according to habitual physical activity level Cox regression models estimated the survivor function adjusted for sex, social class, geographic area, smoking status anxiety/depression at baseline and season of interview. Physical activity categories indicate the number of self-reported 20 min bouts of moderate/vigorous activity per month: Inactive = 0; Low = 1–14 bouts; Moderate = 15–29; Active = 30+ bouts per month

A linear trend across physical activity categories did not adequately describe the association between activity bout and mortality risk (departure from linear trend test: χ_3_^2^ = 10.1, *p* = 0.018). A restricted cubic spline fit to the data revealed that the incidence of all-cause mortality was not a linear function of activity bout; spline covariates were significantly different from zero (Supplementary Figure 1: χ_2_^2^ = 413, *p* ≤ 0.0001). Mortality rates (95 % CI) were highest among individuals who were inactive (no activity bouts per month) at 42.53 (38.94–46.46) per 1,000 PYR, and lowest among those who were meeting guideline activity levels at 6.04 (4.82–7.56) per 1,000 PYR The biggest difference in mortality rates across physical activity categories were between those individuals who were inactive compared to all other activity categories (Table 2). Compared with being inactive, engaging in any episodes of activity was associated with a lower mortality rate over 22.9 years of follow-up, adjusting for age, sex, social class, smoking status, geographical area, self-report of anxiety/depression and season of interview (Table 2, Model 2). Achieving guideline activity levels was associated with a 25 % lower mortality rate in adjusted models (HR, 95 % CI 0.75, 0.58–0.97), compared with being inactive. The inverse association between activity bouts and mortality was observed even for those achieving less than the recommended levels (Table 2, Model 2). Figure 1 shows the inverse association between activity bouts and mortality. As the majority of deaths occurred in the over 50s (1,047/1,175, 90 %), the modelled survival probability for different categories of habitual physical activity is displayed in those aged 50 and over for clarity, adjusting for all known measured confounders (Model 2). The association between habitual physical activity level and mortality held, regardless of cause of death; higher activity levels were inversely associated with risk of death from cardiovascular disease, cancer or other causes (data not shown).

**Table 2:** Hazard ratios (95 % CI) for all-cause mortality by habitual physical activity level; the Allied Dunbar National Fitness Survey (n = 3,912, 1990–2013)

Habitual physical activity^a^	Deaths (N)	Per 1,000 years at risk	Rate per 1,000 person years at risk (95 % CI)	Model 1 (n = 4,301)		Model 2 (n = 3,975)	
				Hazard ratio (95 % CI)	*p*	Hazard ratio (95 % CI)	*p*
Inactive	492	11.57	42.53 (38.94–46.46)	1	0.002	1	0.004
Low	460	36.42	12.63 (11.53–13.84)	0.78 (0.68–0.89)		0.79 (0.69–0.90)	
Moderate	147	16.58	8.86 (7.54–10.42)	0.79 (0.65–0.96)		0.81 (0.66–0.98)	
Active	76	12.59	6.04 (4.82–7.56)	0.74 (0.57–0.96)		0.75 (0.58–0.97)	

Hazard ratios represent the odds of mortality per categorical increase in activity exposure. Model 1 adjusts for age and sex and smoking status. Model 2, as for Model 1, with additional adjustment for social class, geographical area, anxiety/depression at baseline, season of interview

^a^Categories based on no. of bouts of past-month moderate/vigorous activity episodes ≥20 min, where inactive = 0; low active = 1–14; moderate active = 15–29 and active = 30+ bouts per month

In terms of lifetime physical activity, a linear trend did not adequately describe the association between lifetime activity and mortality risk (departure from linear trend test: χ_3_^2^ = 11.7, *p* = 0.008). There was a trend whereby the higher the proportion of life spent actively engaged in sports/exercise, the lower the mortality risk but there was uncertainty about these estimates (for example, adjusted HR, 95 % CI for people spending <25 % their lifetime engaged in regular activities compared to those spending ≥75 % of their adult life active: 0.90, 0.73–1.10).

Likelihood ratio tests showed no evidence of interaction between any of the physical activity measures and age, socioeconomic status or sex (all *p* > 0.05; data not shown). Including baseline BMI in the final model had negligible effects on the association between physical activity and mortality (<8 % change in HRs in models with and without BMI; data not shown). However, BMI data was not available for the full cohort (n = 2,708/3,918). Sensitivity analyses excluding those with a self-report of stroke, heart attack, cancer or diabetes at baseline (n = 321), who died within 5 years of interview (n = 31), or who were underweight (BMI < 18.5 kg/m^2^, n = 42) slightly altered the magnitude of association between activity bout and mortality risk, but in all cases the direction and strength of associations are consistent and our findings were not qualitatively altered (for example, adjusted HR, 95 % CI for active compared to inactive individuals: 0.77, 0.59–1.00). Sensitivity analyses censoring individuals at date of first emigration (n = 145) did not qualitatively change the association between activity and the rate of all-cause mortality (adjusted HR, 95 % CI for active compared to inactive individuals: 0.72, 0.55–0.97). Using time-in-study as the underlying time-scale in Cox models did not qualitatively alter our results (data not shown).

### Population mortality benefits of an active life (Table 3)

Table 3 shows the population-attributable fraction (PAF) for achieving less than guideline activity levels, (here being low or moderately active and engaging in between 1 and 14 or 15 and 29 bouts per month, respectively) as well as for achieving recommended activity levels (30+ bouts), both for the population as a whole and for the sub-group of inactive individuals. Assuming the association between inactivity and mortality is causal, the PAF for all-cause mortality per categorical increase in physical activity level emphasises the significant population mortality benefits of an active life. 20.6 % (95 % CI 6.9–32.3) of population all-cause mortality can be attributed to not meeting recommended activity levels. This attributable fraction is 27.9 (95 % CI 9.6–42.5 %) among the sub-group of inactive individuals. If everyone achieved even low or moderate levels of physical activity, a substantial proportion of premature deaths could be prevented in the population as a whole and in inactive individuals in particular (Table 3). The PAF for all-cause mortality per categorical increase in physical activity level were similar regardless of sex, supporting the significant population mortality benefits of an active life for both men and women (see sex-stratified PAFs in Supplementary Table 2).

**Table 3:** The proportion of deaths that might be prevented if all participants achieved at least the physical activity level indicated, the Allied Dunbar National Fitness Survey

Activity category^a^	Population attributable fraction % (95 % CI)	
	Whole population^b^	Inactive population^c^
Low	8.9 (4.2–13.4)	21.4 (10.2–31.1)
Moderate	15.1 (5.7–23.6)	24.1 (9.3–36.5)
Active	20.6 (6.9–32.3)	27.9 (9.6–42.5)

Adjusted for age, sex, social class, marital status, health authority, season, alcohol intake and smoking status

Assuming causality between physical activity and mortality, PAFs show the percentage of deaths that might be prevented if all participants achieved at least the physical activity level indicated

^a^The number of 20 min bouts of moderate/vigorous activity per month, where low: at least 1; moderate: at least 15; active: at least 30 ‘bouts’

^b^The proportion of deaths that might be prevented if all participants achieved at least the physical activity level indicated

^c^The proportion of deaths of inactive individuals that might be prevented if all inactive participants achieved the physical activity level indicated

## Discussion

In this large population-based prospective UK cohort of 3,918 individuals, meeting activity guidelines of 150 min of at least moderate intensity activity per week, equivalent here to 30+ past-month physical activity bouts, was associated with 25 % lower mortality rate compared to inactive individuals over a median follow-up time of 22.9 years. The largest reduction in risk occurred between the most inactive group (no 20-min bouts of at least moderate activity over the past month) and those achieving the recommended activity levels (30+ activity bouts per month). Although adhering to activity guidelines was associated with substantial mortality benefits, we also show that engaging in *any* bouts of at least moderate intensity activity is better than none, with the hazard of mortality reduced by approximately 20 % in those achieving low or moderate activity levels compared to inactive individuals. These benefits are apparent for both men and women, of all ages and across all socioeconomic groups. Within the total study population, we estimate that 20.6 % (PAF) of all premature deaths might be avoided if everyone achieved the recommended activity levels, after adjusting for known measured confounders. A substantial proportion of premature deaths could be prevented in the population if everyone achieved even low or moderate levels of physical activity. This emphasises the importance of encouraging population-wide increases in activity for the population as a whole, and for inactive individuals in particular. This study confirms the mortality benefits of current activity guidelines that endorse 150 min of at least moderate activity a week, but suggests that engaging in *any* 20 min bouts of activity has beneficial effects on longevity.

### Comparison with prior research

Numerous expert groups have published consensus recommendations endorsing the health benefits conferred by engaging in a weekly minimum of 150 min of moderate to vigorous activity [3–6]. Although several studies have examined the association between physical activity and mortality (for a recent meta-analysis see [28]), few have directly assessed the association of meeting recommended activity levels and mortality due to difficulties in assessing the duration, intensity and frequency of activity across different domains and activity types. Indeed, to our knowledge, only two previous studies have directly quantified the mortality benefits of meeting activity guidelines (150 min of at least moderate activity per week) and both studies focused on the domain of leisure time activity only [10, 11]. One large population-based prospective study found that meeting activity guidelines was associated with a 27 % lower risk of death [10], similar to the protective effect of activity found in this study. Another smaller prospective observational study reported a mortality benefit of meeting activity guidelines in women only [11]. It is possible that these risk estimates were biased due to the non-representative study population, the sole focus on leisure time activity to the exclusion of other activity domains and failure to collect information on activity intensity, which may have led to misclassification. Here, by assessing the total frequency of activity bouts with a minimum intensity and duration across all activity types (sports and recreation, getting about, home activities and occupation) in a large population-based UK sample, the exposure variable may be more precise and hence reported risk estimates more robust. In terms of estimating the burden of premature mortality in the UK that could be eliminated if everyone was active, our results are in line with a recent study which used standardised survey data and estimated the PAF for all-cause mortality associated with inactivity to be 16.9 % [2]. Our use of three categories of physical activity enables PAF estimates associated with a range of activity levels to be assessed, which likely provides more accurate PAF estimates than a binary activity exposure [2]. Overall, this study confirms the mortality benefits of meeting current activity guidelines and, we expand on previous research by showing that engaging in any number of moderate activity bouts on a monthly basis is associated with reduced mortality, consistent with both a dose–response association between activity and mortality [28, 29] and with the idea that there are mortality benefits associated with simply not being inactive [30].

### Strengths and limitations

This large, prospective study in a representative UK population included detailed assessment of activity across different domains and time frames. There was long-term follow-up (median 22.9 years), a high participant response rate (76 %) and the population was socio-economically diverse, ensuring generalizability to similar populations. The use of self-reported physical activity and alcohol data could introduce some measurement error. However any misclassification, if introduced, is likely to be non-differential, which can have complex effects on estimates depending on the particular form of non-differential misclassification [31]. Since younger people were marginally underrepresented in the sample, selection bias may have led to a slight overestimation of the association. However, differences were small, and the survey sample was representative of the age and sex distribution of the general English population at that time [12, 13]. Due to the design of the original ADNFS study, data on some potential confounders was not available and there is a possibility of residual confounding. Known clinical risk factors for mortality—for example, blood pressure and dyslipidaemia—are likely to lie on the causal pathway between physical activity and mortality and as such, it was not appropriate to adjust for these risk factors in our analysis. Data limitations due to the ADNFS study design also precluded our ability to examine the importance of number of days of physical activity practice or time spent sedentary. As exposure data was only collected at the time of the baseline interview, it was not possible to adjust for the effect of time varying covariates. A limitation of the present work is that our conclusions are limited to bouts of moderate-to-vigorous activity lasting 20 min or longer, which is due to the nature of the questionnaire and derived summary measures in 1990. It is possible that health benefits may be achieved by short bouts of high intensity activity [32] or even with lower intensity bouts [30]. Contemporary methods of assessing physical activity objectively will include the full range of intensity and bout duration in the measurement scale and, as part of longitudinal studies, will increase the precision of these estimates. To help inform intervention development, data on which aspects of physical activity, for example, sedentary time or duration of vigorous activity, are associated with particular endpoints should also be included in future research. However, such designs are costly and time-consuming and will take several years to collect data on their association with mortality.

### Clinical and public health implications

Physical activity has numerous beneficial physiologic impacts on the cardiovascular, musculoskeletal, metabolic, endocrine, and immune systems [3]. A systematic review found that individually tailored activity interventions targeting the most sedentary or those most motivated to change their inactive behaviour, were associated with an increase in weekly walking of up to 1 h [33]. A Cochrane Review of trials of physical activity promotion interventions concluded that such interventions increased self-reported activity and fitness [34]. Our data show potential mortality benefits of population-wide achievement of current activity guidelines. The current and longstanding challenge is to develop intervention programmes and public health campaigns [35] which successfully promote the achievement and maintenance of physical activity goals in sedentary populations.

More than half of UK [8] and US adults [3, 36] do not meet minimum activity recommendations. In this study, 85 % did not achieve recommended activity levels. Our findings suggest that adhering to activity guidelines can greatly reduce population all-cause mortality. The attributable fractions for all-cause mortality per incremental increase in physical activity bout frequency emphasise the dose–response relationship between activity and mortality and the potential for considerable health gains. Inactive individuals who report no 20-min bouts of moderate activity per month would experience the largest reduction in mortality risk by achieving the recommended levels; 27.9 %. The use of simple self-reported physical activity questionnaires in clinical practice might be one way of identifying inactive individuals who may benefit most from interventions to increase activity. However, as so few people meet guidelines, our findings suggest that strategies which result in population-wide increases in activity will also be required to achieve reductions in premature mortality [37]. Such upward shifts in the whole population distribution of physical activity will require policy changes to redesign activity into everyday lives [35]. Health promotion efforts should continue efforts to encourage everyone to achieve guideline activity levels, but even modest shifts in the population distribution of physical activity are desirable. Achieving these goals is likely to require interventions targeting collective and individual determinants of physical activity [33, 38].

## Electronic supplementary material

Below is the link to the electronic supplementary material.

Supplementary material 1 (DOCX 179 kb)
