# Supplementary material for: Mortality benefits of population-wide adherence to national physical activity guidelines: a prospective cohort study
Source: Eur J Epidemiol. 2014 Nov 7;30(1):71–9. doi: 10.1007/s10654-014-9965-5 (PMC4356894; doi:10.1007/s10654-014-9965-5)
Supplement: Supplementary file 1 — Supplementary material 1 (DOCX 179 kb) [file 10654_2014_9965_MOESM1_ESM.docx]

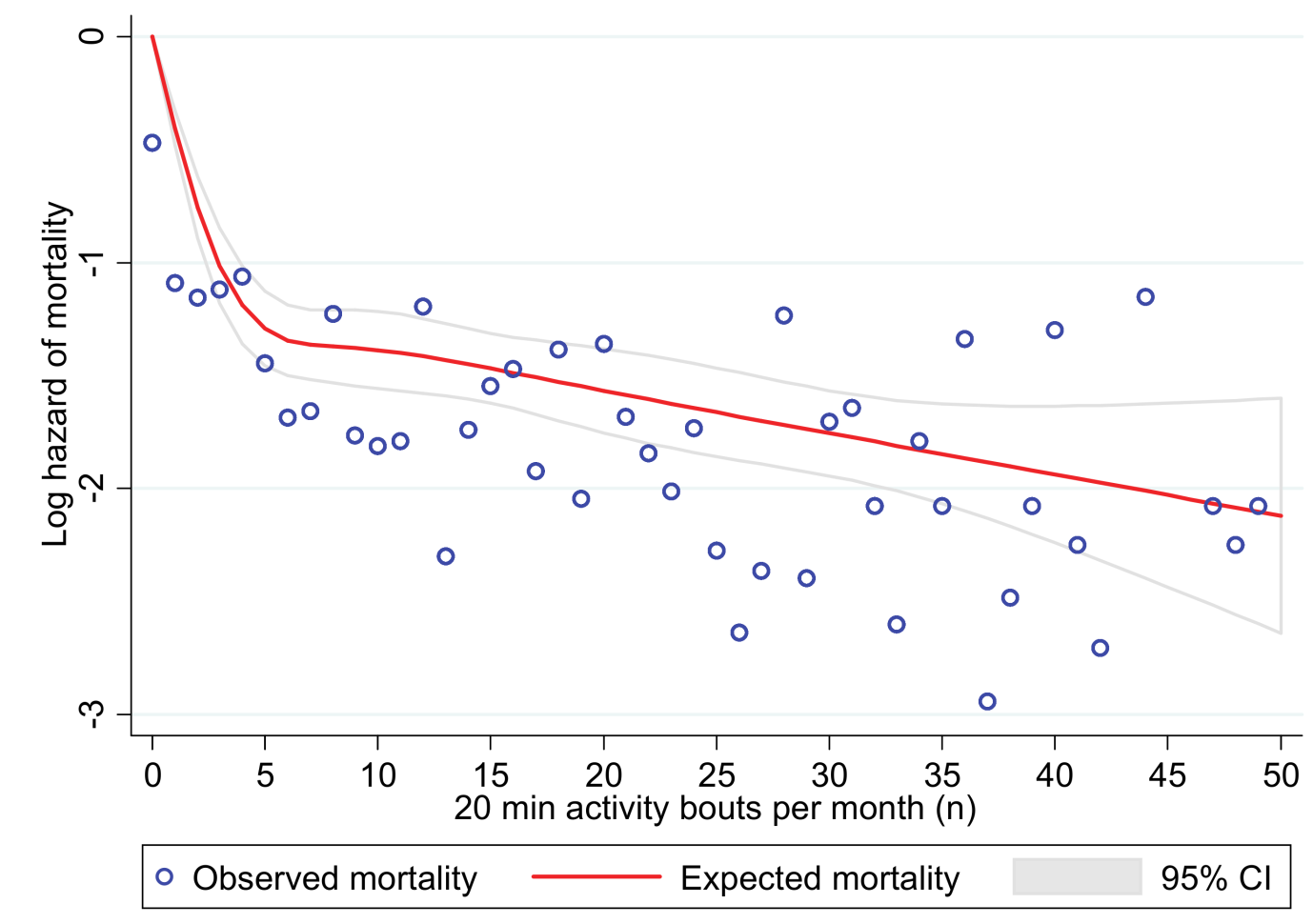


**Supplementary Figure 1: Probability of mortality by number of physical activity bouts in 3918 Allied Dunbar National Fitness Survey participants.** Estimates are from a Cox regression model of all-cause mortality over 22 years of follow-up against a restricted cubic spline function of activity bout at baseline interview; number of 20-minute episodes of moderate to vigorous activity in the past month. The red line plots the probability of death at a given activity bout, with upper and lower 95% CI as a range plot with grey area shading. Open blue circles represent the proportion of deaths observed for the activity bout number indicated.

| **Supplementary Table 1**: Cause-specific mortality over two decades of follow-up in Allied Dunbar National Fitness Survey (n=3918, 1990 to 2013) | | |
| --- | --- | --- |
| Cause of Death | N | Percent |
| CVD | 496 | 42.2 |
| Cancer | 316 | 26.9 |
| Suicide/Violence/ Accident | 22 | 1.9 |
| Other | 341 | 29.0 |
| Death classifications were defined by ICD-10 codes in the range: I00–I99 for CVD deaths; C00–D48 for cancer deaths and V01-Y98 for suicide/ violence/ accidental deaths. | | |

| **Supplementary Table 2. The proportion of deaths that might be prevented if all participants achieved at least the physical activity level indicated, stratified by sex. Data taken from the Allied Dunbar National Fitness Survey** | | |
| --- | --- | --- |
|  | **Population attributable fraction % (95% CI)** | |
| **Activity Category^a^** | **Whole Population^b^** | **Inactive Population^c^** |
| **Male** | | |
| Low | 7.6 (1.1 to 13.7) | 19.6 (0.3 to 33.6) |
| Moderate | 14.4 (2.0 to 25.2) | 24.3 (4.5 to 40.5) |
| Active | 19.2 (1.6 to 33.7) | 27.2 (2.3 to 45.8) |
| **Female** | | |
| Low | 9.9 (3.1 to 16.3) | 22.4 (7.2 to 35.1) |
| Moderate | 16.2 (1.6 to 28.6) | 24.6 (2.5 to 41.6) |
| Active | 23.6 (1.7 to 40.6) | 30.6 (2.3 to 50.7) |

All PAFs adjusted for age, sex, social class, marital status, health authority, season, alcohol intake and smoking status.

Assuming a causal link between physical activity and mortality, PAFs show the percentage of deaths that might be prevented if all participants achieved at least the physical activity level indicated.

^a^ The number of 20 minute bouts of moderate/vigorous activity per month, where low: at least 1; moderate: at least 15; active: at least 30 ‘bouts’.

^b^ The proportion of deaths that might be prevented if all participants achieved at least the physical activity level indicated.

^c^ The proportion of deaths of inactive individuals that might be prevented if all inactive participants achieved the physical activity level indicated.
